# Supplementary material for: Spontaneous formation and relaxation of spin domains in antiferromagnetic spin-1 condensates
Source: Nat Commun. 2019 Mar 29;10:1422. doi: 10.1038/s41467-019-08505-6 (PMC6441100; doi:10.1038/s41467-019-08505-6)
Supplement: Supplementary file 1 — Supplementary Information [file 41467_2019_8505_MOESM1_ESM.pdf]

Reviewers' comments:

Reviewer #1 (Remarks to the Author):

The authors of the present paper experimentally investigate static and dynamic properties of a spin-1 Bose-Einstein condensate of 23Na atoms. The ground-state phase of this system depends on the magnetic field, atomic density, and total magnetization. Since the atomic density and total magnetization have spatial dependence in a trapping potential, the spatial distribution of this system exhibits patterns even if the magnetic field is uniform. The authors control the uniform magnetic field with high precision, and realize these ground-state patterns in a well controlled manner. They also study nonequilibrium relaxation dynamics of this system.

In my view, this paper can be published in Nature Communications. Although the ground-state properties of the spin-1 BEC is well known and the ground states in magnetic-field gradient have already been studied in Ref. 30, the realization of the ground states in uniform magnetic field and its relaxation dynamics will be important for researchers in the field of spinor BECs.

I have some questions and comments, which should be addressed before the publication.

1) In the abstract, the authors mention about the itinerant antiferromagnetism of electron systems, and state that the present phenomenon is magnetic ordering of itinerant bosons. However, it is not clear how these systems are closely related with each other, which is not mentioned in the main text.

2) In Fig. 5, the authors discuss the relation with tunneling, but the definition of energy barrier is unclear. In fact, in the course of the relaxation (at t=1.8s), the mF=1 and mF=0 components overlap with each other, which implies that the barrier is circumvented. The role of spin-exchanging collisions is also unclear. This dynamics might be related with that in PRA 93, 033615 (2016).

3) The GP equation is reduced to 1D assuming that the transverse wave function is the harmonic oscillator ground state. The validity of this approximation is doubtful for \mu \sim \hbar \omega_\perp.

4) Can the relaxation dynamics in Fig. 5 be reproduced with the GP simulation?

5) There is a typo in Fig. 4 (Fig. 4c in the caption is absent).

6) In the title, I do not understand the precise meaning of "quasi-condensates". Does it mean quasi-1D condensates?

Reviewer #2 (Remarks to the Author):

In this manuscript, the authors report on their experiments studying a F=1 spinor BEC in a homogeneous external magnetic field. The paper presents and carefully analyzes both steady-state and time-evolution of the system.

The paper is extremely clear and well written and a pleasure to read. From the accessible introduction, which puts the present work into context with the broader field, to the clear summary of the theory background, to the extremely careful and transparent discussion of the results, I have (almost) nothing to criticize. Some small comments are below. I highly recommend publication of this paper in

Nature Comm.!

1) The one paragraph that I found a bit harder to follow and less clear is the "spatial structure of a trapped system". Maybe it would already be enough to give a short summary of the conclusions at the beginning or the end of this section. As it is now, one reads about the different approaches and then gets told about their short-comings and only learns about the (likely) reasion in the next section.

2) One question that came up in particular when reading the conclusion and the discussion about the role of thermal fluctuations: is there any possibility that quantum fluctuations, which should even be enhanced in 1d, are relevant, as has been recently observed for dipolar quantum gases? Those experiments of course differ in the strength and character of the magnetic interaction, but could a similar beyond-mean-field approach be relevant here?

3) This is a purely technical question: when reading about the very precise magnetic field compensation described in the methods, I was wondering if the authors have considered an active field cancellation? As I understand the methods, the external fields are compensated on the mG level once by radio-frequency spectroscopy. An active field measurement and compensation at this level is technically feasible, but maybe not required?

Reviewer #3 (Remarks to the Author):

GENERAL COMMENTS/REMARKS

Jiménez-Garcia, Invernizzi and coworkers report on a study of a spinorial system of sodium atoms in their lowest hyperfine state F=1. The authors provide a clear and elegant picture of such a complex system, both introducing a simple model to describe it from a theoretical point of view and reporting direct experimental results.

The work is new and useful to anyone working on multicomponent systems that can undergo phase separation.

The manuscript is written in a very clear way, really instructive and elegant, as a good textbook should be. The data have been treated in a rigorous way and a statistical analysis was reported. A few minor English mistakes are present and I will point them out at the end of this report.

I find their study very complete. The authors are able to introduce a problem in a simple way and then extend it to more realistic or general cases describing the effects of each new feature under consideration. In particular they illustrate the spin-1 system in the uniform case and identify 3 distinct phases characterized by the magnetization. The authors extend the description to the harmonically trapped case, where the 3 phases can coexist (in different regions) and report measurement in a quasi-1d trap studying the axial density distribution. First calculations are presented in the local density approximation and then the authors estimate the effect of beyond-LDA terms. They study the case at T=0 and then introduce thermal effects.

Equilibrium configurations are depicted and then they study how such configurations are reached when the system is initially prepared in a strongly out-of-equilibrium state.

The experimental details are described in a very extensive way (considering main text and methods), therefore I believe that other groups can reproduce the experiment and obtain similar results. I

suggest, however, to add a few more details regarding the cooling ramping times to understand how the spinor BEC is normally created, and convince the reader that the BEC is created in a spinorial form since the beginning, and not after some evolution time nor dynamically during the evaporation.

The parameter control (magnetic field, magnetic field gradient, trap frequencies, dimensionality, temperature) is high and well described. However, I think that the authors should better describe how the state-selective imaging was implemented. How well can they measure the number of atoms in the different states? Do they adopt any specific method to avoid counting problems?

SPECIFIC COMMENTS/REMARKS

1) Please clarify better what m_|| is, at least giving a few simple examples of differently magnetized systems with the corresponding m_II values.

2) In order to avoid misunderstanding I suggest not to use the symbol (parallel) for the magnetization along the magnetic field (y) and the symbol (perpendicular) for the radial, transverse frequency (y,z). Also the word "longitudinal" can produce some confusion. The reader might think alternatively to the long axis of the condensate (x) or to the magnetic field direction (y). I think that the authors can make a better and unambiguous choice for these variables that clearly separates the geometrical coordinates (preferential axis x) from the magnetic field (preferential axis y).

3) Figure 1b shows the integrated equilibrium density distributions in each of the 3 states and for 3 different magnetic fields. I think that it would be better to move the color/line codes for m_F on one side (since it is valid for all 6 plots) and write the magnetic field B=30, 70 and 150 mG above the corresponding density distributions.

4) Figure 2a shows q/h on the vertical axis. For helping readers that are not familiar with sodium atoms, I suggest to add the corresponding absolute magnetic field values (at least the minimum and the maximum values of the reported range) on the right side of the figure.

5) Page 4, first column, top. "The interaction Hamiltonian conserves the ... field B". Down to what q (and B) is this still valid?

6) Page 6, second column. Clarify better the expression "the domain wall becomes steep". The domain wall is a surface.

TYPOS
Abstract.
1) a system of localized spins with ferromagnetic interactions align themselves to a common direction -> localized spins with ferromagnetic interactions align themselves along a common direction
2) i.e. in the absence of external force -> i.e., in the absence of external forces
3) prepared out-of-equilibrium -> prepared out of equilibrium

Introduction.
4) an hyperfine -> a hyperfine
5) to compensate magnetic field gradients -> to ompensate for magnetic field gradients
6) where transverse motion is almost frozen -> where the transverse motion is almost frozen
7) Page 6: immmiscible -> immiscible
8) Page 6: does not completely vanishes -> does not completely vanish
9) Page 9: a distributions -> a distribution

OTHER MINOR NOTES
1) Please specify the exact references at page 2, first column ("see [37] and below "). What is "below"?
2) Page 3, first column. (1d) is already defined on page 1.
3) Page 3, first column. When talking about measured 1/e lifetime, do they refer to the total atom number decay or to what else? Please specify.
4) Page 9, second coulmn. After achieving a degenerate Bose gas -> After achieving a degenerate spinor Bose gas.
5) Update reference 13, that in the meantime was published.

FINAL COMMENT
After the authors have addressed the comments reported above, I think the manuscript can be published in Nature Communications.

Detailed response to Reviewer #1:

1) In the abstract, the authors mention about the itinerant antiferromagnetism of electron systems, and state that the present phenomenon is magnetic ordering of itinerant bosons. However, it is not clear how these systems are closely related with each other, which is not mentioned in the main text.

The point of the introduction is to emphasize physically relevant with magnetic order parameters that are not spatially uniform. We give the example of the stripe phase in highly correlated materials to illustrate that it is a rich and broad field, in fact with many more possibilities than "simple" spin problems. We unfortunately have no space to explain this in more details neither in the abstract nor in the Introduction. In fact, we had to shorten the abstract to fit into the 150 words limit set by Nature Communications. We opted to extend the Discussion section with a more detailed explanation replacing the first paragraph of that Section:

Magnetically ordered many-body systems characterized by non-uniform order parameters are plentiful. The simplest example corresponds to antiferromagnetism of localized spins (here the adjective ``localized'' means that the mobility of the spin-carrying particles is irrelevant, and that the physics reduces to a pure spin problem). The problem becomes substantially richer if the spin-carrying particles are mobile (``itinerant magnetism''). The stripe phases of electrons in strongly correlated materials are a prominent example. These materials are hole-doped antiferromagnets, which organize for certain doping levels in antiferromagnetic domains separated by nanometer-size conducting domain walls. This structure is believed to arise from the competition between the kinetic energy of the holes and the exchange interactions between the spins. This example illustrates that one can expect (and often finds) non-uniform phenomena in the magnetism of itinerant quantum particles. In this article, we have investigated the formation and relaxation of inhomogeneous spin domains in a quasi-1d spin $F=1$ Bose gas with antiferromagnetic interactions. The low-field configuration is a mixed phase of the $m_F=\pm 1$ components stabilized by antiferromagnetic spin-exchange interactions. An applied uniform bias magnetic field favors the appearance of $m_F=0$ atoms through the QZE shift. The two influences compete agains each other, and this competition leads to a critical value of the QZE above which $m_F=0$ atoms appear and spontaneously organize in a spin domain near the trap center. We experimentally measured the critical value $q_{1,\textrm{exp}}$ where a $m_F=0$ domain appears and characterized the phase diagram in details.

2) In Fig. 5, the authors discuss the relation with tunneling, but the definition of energy barrier is unclear. In fact, in the course of the relaxation (at t=1.8s), the mF=1 and mF=0 components overlap with each other, which implies that the barrier is circumvented. The role of spin-exchanging collisions is also unclear. This dynamics might be related with that in PRA 93, 033615 (2016).

The picture of an energy barrier is applicable at early times, where the two components are clearly separated. We agree with the referee that it does not make a lot of sense for longer times where a substantial population of m_F=0 is in the trap center. However we feel that it is still useful to recall this picture of an effective potential barrier, as it is often used in the literature (for instance, in the reference mentioned by the referee) and provides a simple (but, as we show, incomplete) scenario for relaxation.

We respectfully disagree with the referee that the role of spin-changing collisions is unclear. In fact, we believe our results show in a convincing way that the relative motion of the Zeeman components is governed by them (Figure 5d). Atoms in m_F=+/- 1 produced in the interface region can freely migrate inside the m_F=+1 domain, and this provides a mechanism by which m_F=0 atoms can pass through the m_F=+1 component, different from tunnelling over an almost macroscopic distance of tens of microns. We have added a paragraph to explain this mechanism in detail and hopefully convince the referee:

The process is most likely dominated by excitations residing initially in the inferface between the $m_F=0$ and $m_F=+1$ regions, and seeding the long-time dynamics\,\cite{stamperkurn1999b}. The effective mean-field potential experienced by atoms in $m_F=-1$ inside the volume of the $m_F=+1$ domain is not exactly flat but slightly attractive, $V_{\textrm{eff}}(x)=\frac{1}{2}M_{\textrm{Na}}x^2+\overline{g}\rho(x)-g_s \rho_{+1}(x) \approx \mu-g_s \rho_{+1}(x)$. Atoms in $m_F=-1$ created in the interface can thus freely migrate towards the trap center, where they can ``recombine'' with $m_F=+1$ atoms to generate $m_F=0$ atoms. This mechanism effectively enables $m_F=0$ atoms to "crawl through" the otherwise impenetrable $m_F=+1$ component. This mechanism could be coherent (preserving the initial relative phase), but need not be. Given that $T \gg g_s \rho$, we believe it is likely that the relaxation process is seeded by the thermal component initially present near the interface.

We believe the mechanism at play in our experiments is different from the ones studied in the quoted paper [Eto *et al*. PRA **93**, 033615 (2016)], where two miscible or immiscible hyperfine components are involved without the possibility of spin-changing collisions. The experiment in this paper appears similar at first sight, but the experimental parameters differ by orders of magnitude. They apply a bias field of 11 G which supresses completely spin changing collisions (we use 600 mG), and a gradient of 900 mG/cm (we use 10 mG/cm). It is then not really surprising that we do not observe the bouncing motion reported by Eto *et al.* in the "strongly immiscible" regime, nor the formation of stripes after a few hundred ms that they report (likely due to the conversion of the initial kinetic energy into density modulations). We however agree with the referee that the experimental setup and procedures are similar, and that it is natural to ask whether the same results are obtained in the two experiments. We have added the reference to our paper and a paragraph comparing this work and ours:
A recent experiment by Eto \textit{et al.}\cite{eto2016a} appears similar at first sight, but reports drastically different results: Bouncing of the spin components against each other in the strongly immiscible regime, followed by relaxation in a few hundreds of ms to a highly excited state with large kinetic energy. We believe the mechanism at play in our experiments is different from the ones studied in \cite{eto2016a}, where the experimental parameters differ by order of magnitudes. They apply a bias field of 11 G which supresses completely spin changing collisions (we use 600 mG), and a gradient of $900\,$mG/cm (we use $10\,$mG/cm). It is then not really surprising that we do not observe the bouncing motion, nor the formation of stripes reported by Eto \textit{et al.} in the ``strongly immiscible'' regime.

3) The GP equation is reduced to 1D assuming that the transverse wave function is the harmonic oscillator ground state. The validity of this approximation is doubtful for \mu \sim \hbar \omega_\perp.

We agree with the referee that the 1D simulation is not reliable to reproduce physics involving energies comparable to the transverse oscillator energy, e.g. dynamics experiments where sizeable transverse motion can occur. In the experiments reported in our paper, there is no such motion due to the low energies and long timescales involved. What matters is the spin healing length, which is about ten times larger than the transverse size R of the condensate. As a result, a transverse variation of the spin density costs an

energy ~1/R^2, on the order of 100 g_s n. This is prohibitive and explains why the spin density only depends on the longitudinal coordinate to a very good approximation. In that situation the spin physics can be described by an effective 1d equation after integrating out the transverse coordinates. The differences between the aforementioned approach and the purely 1d GP approach are small (see the comparison between the density profiles in the SM: differences are in the 10 % range). Essentially the coupling constant g_1d is renormalized and slightly lower than the pure 1d value. Substantial deviations in the density profile are only visible for \mu >= \hbar\omega_\perp, *i.e.* outside of the regime we explore in our work. For that reason, we opted for the simpler, strictly 1d approach for the numerical calculations.

4) Can the relaxation dynamics in Fig. 5 be reproduced with the GP simulation?

The GP simulation performed at T=0 does not reproduce the relaxation dynamics. In fact, it shows almost no dynamics. This is expected since a seed (from quantum or classical thermal fluctuations) must be added to trigger relaxation. There is no such seed in the standard T=0 GP equation.

5) There is a typo in Fig. 4 (Fig. 4c in the caption is absent).

Corrected, thank you.

6) In the title, I do not understand the precise meaning of "quasi-condensates". Does it mean quasi-1D condensates?

A quasi-condensate denotes a bosonic gas which is almost indistinguishable from a true condensate except for its phase coherence properties. In a true condensate, the coherence length is infinite ("off-diagonal long-range order"). Phase coherence extends over the entire system, which justifies that we describe it with a macroscopic wavefunction. In a quasi-condensate, due to fluctuations of the phase the coherence length is shorter than the system size (but longer than for a normal gas as the same temperature). However, density fluctuations are supressed as in a true condensate, so that the system can be described by a classical field with constant amplitude (density) but fluctuating phase. While this certainly applies to our experiment, this is not a central point. We removed the "quasi-" from the title and only mention it in the corresponding paragraph, with more references and a slightly expanded description of quasi-condensates:

Because of its quasi-1d character, the bosonic quantum gases studied in our experiment are quasi-condensates [REFS}]: Density fluctuations are essentially frozen, as for a true condensate, but phase fluctuations along the weak axis of the trap can be substantial at finite temperatures. These phase fluctuations do not affect the thermodynamic properties of the mixture, but they show up as density stripes in time-of-flight images [REFS].

Detailed response to Reviewer #2:

1) The one paragraph that I found a bit harder to follow and less clear is the "spatial structure of a trapped system". Maybe it would already be enough to give a short summary of the conclusions at the beginning or the end of this section. As it is now, one reads about the different approaches and then gets told about their short-comings and only learns about the (likely) reasion in the next section.

We added a paragraph at the end of the Section summarizing our findings and introduce the next one:

To summarize, we used the local density approximation to describe the spatial structure of a trapped gas in terms of the phase diagram of the uniform system at $T=0$. We were able to account qualitatively for the observations, but found quantitative differences. In particular, the measured critical field where spin domains appear is higher than predicted. In the next Section, we show that the discrepancy between the measured q_1 and the $T=0$ prediction, as well as the difficulty in identifying q_2 in experiments, can be understood qualitatively by considering the role of a finite temperature of the sample, which is the topic of the next Section.

2) One question that came up in particular when reading the conclusion and the discussion about the role of thermal fluctuations: is there any possibility that quantum fluctuations, which should even be enhanced in 1d, are relevant, as has been recently observed for dipolar quantum gases? Those experiments of course differ in the strength and character of the magnetic interaction, but could a similar beyond-mean-field approach be relevant here?

In principle, the referee is correct to stress that quantum fluctuations could be playing the same role as thermal fluctuations. They are enhanced in 1d in comparison with 3d, but so are thermal fluctuations. In single-component 1d gases and for weak interactions, thermal fluctuations dominate quantum fluctuations when T >> \mu. We can expect a similar conclusion for spin excitations when T >> g_s \rho, which is why we insist on thermal excitations in the main paper. We added a sentence to stress that quantum fluctuations are expected to have a similar (although weaker) effect:

Note that the measured temperature is substantially higher than the spin-dependent energies η, q explored in this work. Hence, even if quantum and thermal fluctuations probably lead to qualitatively similar effects on the domain formation, we expect the latter to be dominant for our experimental conditions.

3) This is a purely technical question: when reading about the very precise magnetic field compensation described in the methods, I was wondering if the authors have considered an active field cancellation? As I understand the methods, the external fields are compensated on the mG level once by radio-frequency spectroscopy. An active field measurement and compensation at this level is technically feasible, but maybe not required?

We have considered (and in fact developed) a system for active stabilization of a uniform bias field below 1 mG (the system actually only stabilizes one component of the magnetic field). This will appear in a joint paper with the university of Hamburg and Birmingham. In the present work, this was not necessary because the static compensation of external fields is in practice stable over months. Active compensation could have been useful to suppress the residual fluctuations along the vertical axis (see discussion in the SM) and push q to lower values than the ones studied in this work. This would not add much to the physics we study here, since the +/-1 mixture remains essentially the same for any q below q_1. One could also envision stabilizing the magnetic gradients as well. This is significantly more complicated, as it requires differential sensors and taking into account the vector nature of the magnetic field.

Detailed response to Reviewer #3:

GENERAL COMMENTS/REMARKS

I suggest, however, to add a few more details regarding the cooling ramping times to understand how the spinor BEC is normally created, and convince the reader that the BEC is created in a spinorial form since the beginning, and not after some evolution time nor dynamically during the evaporation.

We have added to the Section Methods – Optical Dipole Trap:

The spinor gas is held in a crossed dipole trap (CDT) created at the intersection of two Gaussian beams propagating along the x and z axes. We prepare a normal gas with a well-defined magnetization far above the critical temperature using the methods described in details in \cite{jacob2012a,zibold2016a}. We then cool the sample to degeneracy using standard evaporative cooling, and obtain a three-dimensional condensate in the CDT with $>90\,%$ condensate fraction.

[...] I think that the authors should better describe how the state-selective imaging was implemented. How well can they measure the number of atoms in the different states? Do they adopt any specific method to avoid counting problems?

We have added a new section *Stern-Gerlach imaging and image analysis* to the Supplementary Informations to describe the implementation of absorption imaging and how we extract linear profiles from the 2D images.

SPECIFIC COMMENTS/REMARKS

1) Please clarify better what m_|| is, at least giving a few simple examples of differently magnetized systems with the corresponding m_II values.

We have modified the section *Brief Review of Ultracold Spin-1 Gases* according to the suggestion of the referee:
The partial densities are given by $\rho_{m_F}=\vert \Psi_{m_F} \vert^2$, and the total density by $\rho = \sum_{m=0,\pm 1} \rho_m$. [...] We denote by $m_{\|}$ the component of ${\bf m}$ along the axis of the applied magnetic field ${\bf B}$. For instance, for a spin-polarized gas of atoms in $m_F=1$, the magnetization density is equal to the total density, $m_{\|}=\rho$, while for atoms in $m_F=0$ $m_{\|}=0$. [...] The interaction Hamiltonian conserves the total longitudinal magnetization $M_{\|}=\int d{\bf r}\,m_{\|}$.

2) In order to avoid misunderstanding I suggest not to use the symbol (parallel) for the magnetization along the magnetic field (y) and the symbol (perpendicular) for the radial, transverse frequency (y,z). Also the word "longitudinal" can produce some confusion. The reader might think alternatively to the long axis of the condensate (x) or to the magnetic field direction (y). I think that the authors can make a better and unambiguous choice for these variables that clearly separates the geometrical coordinates (preferential axis x) from the magnetic field (preferential axis y).

We actually discussed this point at length when preparing the manuscript. The reason to choose the notation m_|| is that the orientation of the bias magnetic field is not fixed to y (see the Section about cancellation of magnetic forces in the SM), and introducing yet another frame of reference was rather awkward. We used the term "longitudinal

magnetization" to stress the fact that the transverse components of ***m*** are not conserved by the interactions (they can change when spin-changing collisions take place). We understand the concern of the referee that this can potentially lead to confusion for the reader. We renamed the transverse trap frequency to \omega_y, and the adjective "longitudinal" is no longer used. We believe these changes achieve the goal of the referee.

3) Figure 1b shows the integrated equilibrium density distributions in each of the 3 states and for 3 different magnetic fields. I think that it would be better to move the color/line codes for m_F on one side (since it is valid for all 6 plots) and write the magnetic field B=30, 70 and 150 mG above the corresponding density distributions.
We followed the suggestion of the referee and modified Figure 1 accordingly.

4) Figure 2a shows q/h on the vertical axis. For helping readers that are not familiar with sodium atoms, I suggest to add the corresponding absolute magnetic field values (at least the minimum and the maximum values of the reported range) on the right side of the figure.

We followed the suggestion of the referee and modified Figure 2 accordingly.

5) Page 4, first column, top. "The interaction Hamiltonian conserves the ... field B". Down to what q (and B) is this still valid?

The conservation of M_\parallel follows from fundamental symmetries of the interactions. The interactions between the atoms are to a very good degree determined by the van der Waals tails of the interaction potential, i.e. they originate in the electromagnetic interactions involving the two outer electrons of the two alkali atoms and the electronic cores. As a result, the interaction potential is independent of the spin, and without magnetic field the total spin of the atom pair is actually conserved. A magnetic field breaks the full rotational symmetry and only leaves the spin projection on its direction as a conserved quantity. The conclusion is that the conservation of magnetization by interactions is always valid, no matter how small B is, being the consequence of a symmetry of the Hamiltonian. Of course, if other terms are taken into account (dipole-dipole interactions, for instance), this conclusion may be challenged. Dipole-dipole interactions are extremely small for Sodium, about 100 times smaller than the spin-dependent interaction.

6) Page 6, second column. Clarify better the expression "the domain wall becomes steep". The domain wall is a surface.

We meant by "domain wall" a shell of finite thickness, on the order of the spin healing length, rather than a surface. We avoid the term "domain wall" in the revised version and use "interface" instead in all instances. We modified the offending sentence to

This cost comes from the balance between the kinetic energy, increasing when the width of the interface decreases, and the interaction energy, increasing when the interface spreads out due to the increased overlap between the two components.

TYPOS All corrected, thank you !

OTHER MINOR NOTES

1) Please specify the exact references at page 2, first column ("see [37] and below "). What is "below"?

We change the reference to : *(see[37] and Section Spatial Structure of a Trapped System below)*

2) Page 3, first column. (1d) is already defined on page 1.

Corrected

3) Page 3, first column. When talking about measured 1/e lifetime, do they refer to the total atom number decay or to what else? Please specify.

The sentence was rewritten as :

The total atom number decays with a measured $1/e$ lifetime around $50\,$s, presumably limited by residual evaporation and three-body recombination.

4) Page 9, second coulmn. After achieving a degenerate Bose gas -> After achieving a degenerate spinor Bose gas.

modified.

5) Update reference 13, that in the meantime was published

Updated.

Reviewers' comments:

Reviewer #1 (Remarks to the Author):

All the questions and comments in my first report have been adequately addressed in the revised version of the manuscript. I can now recommend this paper for publication in Nature Communications.

Reviewer #2 (Remarks to the Author):

The authors have carefully and thoroughly addressed all comments of all 3 referees, in my opinion. I have no further questions to the authors and recommend publication of the manuscript as it is.

Reviewer #3 (Remarks to the Author):

The authors followed most of the suggestions provided by all referees in the first round and improved the clarity of the manuscript.

I have a few more comments that, in my opinion, could make the paper even clearer

1) I suggest to replace the last panel of figure 2 with the total number of atoms in each state (integrted) as a function of q/h. In this way, if I understand well, one can see that the total magnetization is preserved to 0.5 and the system goes from 0.75% in mf=+1, 0.25% in mf=-1 for low fields, to 0.5% in mf=+1, 0.5% in mf=0 (for large fields).

2) I'm sorry not to have it pointed out this during the first round, but I am a bit confused by the phase diagram reported in Figure 3b. How can the grey area represent Phase I (all atoms in mf=0) for values of magnetization density different from zero? Is the horizontal axis maybe referring to the total magnetization M_|| ?

3) In the method sections, the authors could add one short sentence illustrating how the fixed magnetization is experimentally set.

TYPOS
1) A comma is missing in the last paragraph (red) at the end of page 3, just before "m_II=0.".
2) Correct labelling in the caption of Figure 2. There are four panels in the figure, while the text refers to just two.
3) Page 4, first paragraph: determine -> determines
4) Page 5, Table I: posssible -> possible.

Other than that, I am happy to see this work published on Nature Communications.

Dear Editor,

We would like to resubmit the revised version of our article "*Spontaneous formation and relaxation of spin domains in spin-1 quasi-condensates*". We thank again the referees for their positive appreciation and thorough work. Referee 3 has a few more comments, which we addressed in this new version (details below). We believe that the changes made to the article address the questions raised by the referees.

Thank you and best regards,

Response to Reviewer #3:

1) I suggest to replace the last panel of figure 2 with the total number of atoms in each state (integrted) as a function of q/h. In this way, if I understand well, one can see that the total magnetization is preserved to 0.5 and the system goes from 0.75% in mf=+1, 0.25% in mf=-1 for low fields, to 0.5% in mf=+1, 0.5% in mf=0 (for large fields).

We envsionned the suggestion by the referee in a previous iteration. We felt (and still feel) that plotting the central density is a better measure of the location of the transition, especially at finite temperatures where the contribution of mF=0 atoms below q_1 (for instance) is less pronounced as for the total population. We however added a new supplementary figure S4 showing the populations of the Zeeman states to follow the referee's suggestion and provide the information not included in the main figure 2.

2) I'm sorry not to have it pointed out this during the first round, but I am a bit confused by the phase diagram reported in Figure 3b. How can the grey area represent Phase I (all atoms in mf=0) for values of magnetization density different from zero? Is the horizontal axis maybe referring to the total magnetization M_|| ?

The grey area in the phase diagram of Fig. 3 is in fact avoided as the sytem "jumps" from phase I to phase II for the particular example displayed. We have removed the grey area, which we agree was confusing, and added in the caption of the figure:

For the particular example shown in the figure, phase I is realized near the center of the cloud, and the state of the system jumps from phase I to phase II along the dashed line when the density reaches a critical value.

3) In the method sections, the authors could add one short sentence illustrating how the fixed magnetization is experimentally set.

We added the sentence : *We prepare a normal gas with a well-defined magnetization far above the critical temperature using spin-distillation in an applied magnetic field gradient at the beginning of the evaporation*

TYPOS
1) A comma is missing in the last paragraph (red) at the end of page 3, just before "m_II=0.".
2) Correct labelling in the caption of Figure 2. There are four panels in the figure, while the text refers to just two.

3) Page 4, first paragraph: determine -> determines

4) Page 5, Table I: posssible -> possible.

All corrected, thank you.

REVIEWERS' COMMENTS:

Reviewer #3 (Remarks to the Author):

The authors addressed all residual issues that I raised. The manuscript can be published in Nat Commun.
