# Supplementary material for: Spontaneous formation and relaxation of spin domains in antiferromagnetic spin-1 condensates
Source: Nat Commun. 2019 Mar 29;10:1422. doi: 10.1038/s41467-019-08505-6 (PMC6441100; doi:10.1038/s41467-019-08505-6)
Supplement: Supplementary file 2 — Peer Review File [file 41467_2019_8505_MOESM2_ESM.pdf]

Supplementary Material for:
Spontaneous formation and relaxation of spin domains in spin-1 quasi-condensates

K. Jiménez-García, A. Invernizzi, B. Evrard, C. Frapolli, J. Dalibard and F. Gerbier
Laboratoire Kastler Brossel, Collège de France, CNRS,
ENS-PSL Research University, Sorbonne Université,
11 Place Marcelin Berthelot, 75005 Paris, France
(Dated: December 13, 2018)

SUPPLEMENTARY METHODS

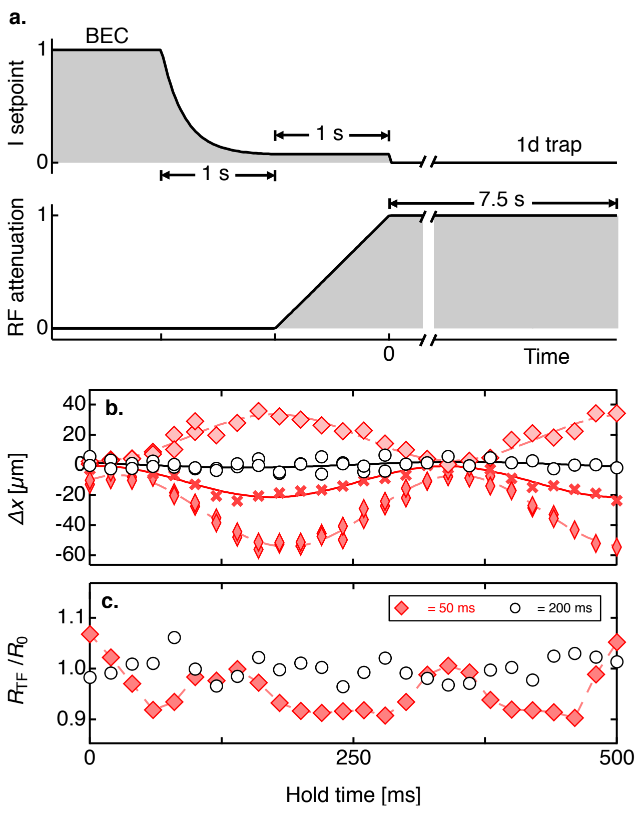

SUPPLEMENTARY FIGURE 1. **Adiabatic transfer to the 1D trap. a.** We adiabatically ramp down the power of the z beam in order to transfer the atoms from the crossed-dipole trap, where the BEC is first produced, to a single beam optical trap. The ramp to turn off the z beam is done is two steps: *i.)* changing the setpoint of the intensity lock (I setpoint), and *ii.)* rf-attenuation (see text). We ensure the adiabaticity of the transfer to 1D by minimizing the amplitude of **b.** center-of-mass and **c.** breathing excitations after the ramps. **b.** The symbols correspond to the relative position Δx of the x beam waist with respect to the crossing of the trapping beams, with $\Delta x \in (-60, 40)\mu$m. The open symbols correspond to the optimum alignment where dipole excitations are minimized. **c.** The symbols correspond to the evolution of the Thomas-Fermi radius corresponding to the fast (closed symbols) and slow (open symbols) removal of the z beam.

Adiabatic transfer into a 1d optical dipole trap: After achieving a degenerate spinor Bose gas in the crossed dipole trap, we adiabatically ramp down the intensity of the z-propagating beam (in short: z beam), leaving the atoms in a single-beam optical dipole trap with measured trap frequencies $(\omega_x, \omega_y, \omega_z) = 2\pi \times (3.11, 268, 268)$ Hz. The intensities of the laser beams creating the dipole traps are stabilized by a servo-loop including an acousto-optical modulator (AOM) to control the laser power to better than one percent. The transfer is highly sensitive to misalignment, in particular to the relative location of x beam waist with respect to the crossing of both beams. We systematically align both beams by minimizing the amplitude of residual dipole oscillations enhanced when the x beam waist is away from the intersection of the trapping beams. Supplementary Figure 1**b** shows dipole oscillations whose amplitude is controlled by the relative position of the beams.

The relevant time scale for adiabatic removal of the z beam is set by the weakest trapping frequency. The removal of the z beam is done in two steps (see Supplementary Figure 1**a**). We first lower the set point of the z beam power from 15 mW down to 1 mW using an exponential ramp of time constant $\tau = 200$ ms. This time scale is sufficiently long compared to the longest trap period in the remaining crossed dipole trap $2\pi/\omega_x \approx 12$ ms to avoid exciting collective modes. After the first ramp, we keep the setpoint of the intensity lock constant (disactivating the servo-loop with a sample-and-hold circuit), and use an auxiliary voltage-controlled attenuator to suppress the radio-frequency (rf) signal driving the z beam AOM. For the experiments shown in Figs. 1, 2 and 4 in the main text, the rf suppression is done in 1 s, with an additional hold time of 7.5 s. For the dynamics experiments shown in Fig. 5, the rf suppression is done in 5 s with no hold time, and a oscillation time variable from 0 to 20 s.

We determine the time scale for adiabaticity judging by the amplitude of breathing oscillations and the extent of axial density fluctuations (manifested as fringes along the density distribution after TOF). For an anisotropic trap with axial symmetry, breathing oscillations are expected to occur at a frequency of $\omega_{\text{breathing}} = \sqrt{5/2}\omega_x \approx 2\pi \times 5$ Hz [1]. Supplementary Figure 1**c** shows the Thomas-Fermi (TF) radius along the weak axis measured as a function of hold time after the removal of the z beam.

The breathing oscillations observed for an intensity ramp with $\tau = 50$ ms are suppressed for a slower ramp with $\tau = 200$ ms.

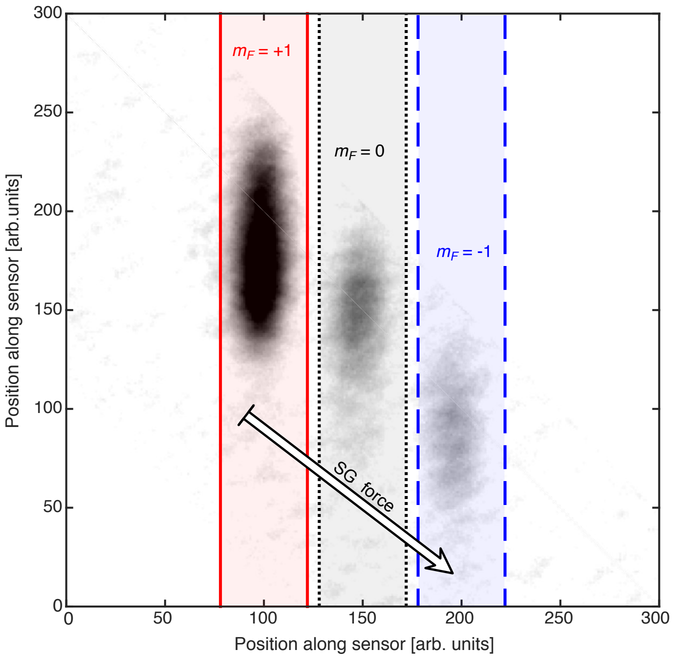

SUPPLEMENTARY FIGURE 2. **Absorption image after Stern-Gerlach expansion.** The shaded areas show the integration boxes used to obtain the linear density profiles.

Stern-Gerlach imaging and image analysis: The sequence used to record absorption images has been described in [2]. We release the atoms by switching off the optical dipole trap, and apply a magnetic force to separate the three Zeeman components using the Stern-Gerlach effect. The force is applied for an expansion time of 8 ms. The spatial distribution observed directly on the charge-coupled-device camera is shown in Supplementary Figure 2. The typical separation between two components ($\sim 110\,\mu$m center-to-center along the Stern-Gerlach separation axis) is larger than the radial extent of the cloud ($20\,\mu$m radius along the y axis) when the image is taken. We isolate the contribution of each component by defining three regions of interest around each cloud using boxes with axis parallel to the weak trap axis x, as shown in the Supplementary Figure 2. Integrating over the perpendicular coordinate for each box we obtain the linear profiles discussed in the article. The absorption cross-sections are slightly different for the three components, because of different Clebsch-Gordan coefficients and a magnetic field present during the image. We calibrate them using the procedure described in [2].

Calibration of applied magnetic field gradients: Supplementary Figure 3**a** shows a sketch of the experimental setup. A pair of gradient coils is used for compensation stray magnetic field gradients and for initiating the relaxation dynamics in our experiments. We calibrate the magnetic field gradient b'_x by measuring the displacement of the center of mass (c.o.m.) of polarized systems $x_{m_F=\pm1} = \pm g_F \mu_B b'_x / m_{\rm Na}\omega_x^2$, which is linearly proportional to the gradient b'_x, where $g_F = -1/2$ is the Landé $g-$factor and μ_B is the Bohr magneton. Our gradient calibration was performed with a bias magnetic

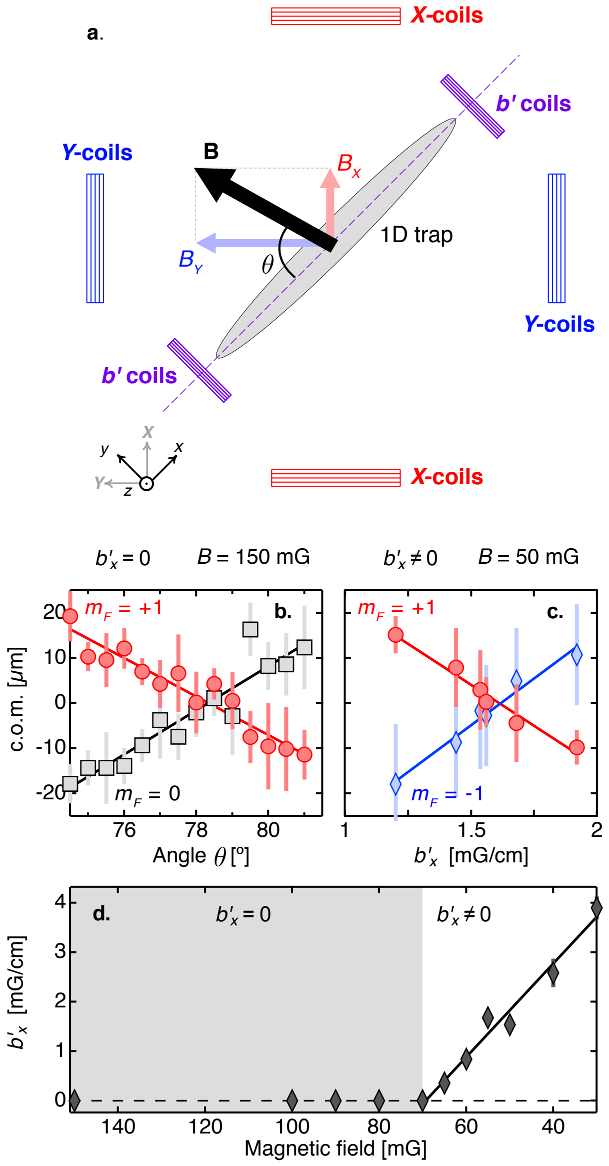

SUPPLEMENTARY FIGURE 3. **Magnetic force cancellation. a.** Experimental setup. We set the amplitude B and the direction θ of the bias magnetic field in the $x - y$ plane using the X- and Y-coils. **b**. Center of mass (c.o.m) of $m_F = 0, +1$ components at $B > 70$ mG, where we cancel magnetic forces by scanning the direction of the magnetic field and $b'_x = 0$ (angle tuning method). **c.** c.o.m. of $m_F = \pm1$ components at $B < 70$ mG where we cancel magnetic forces by applying an external gradient $b'_x \neq 0$ using the gradient coils (direct compensation method). **d.** b'_x as a function of magnetic field. Note that the bias magnetic field decreases from left to right.

field $B = 150$ mG ($\theta = 78°$, see below) and with trap frequencies 3 Hz $< f_x <$ 6 Hz. The experimental result $b'_{x,\,\mathrm{exp}}(I) = I \times 23.9(7)$ mG/cm/A is in excellent agreement with the value expected from a magnetostatics calculation.

Magnetic force cancellation: Our experiments are performed after carefully cancelling stray magnetic gradients along the weak axis of the trap. Assuming that gradients vary slowly in space at the scale of the atomic gas, a multipole expansion leads to a magnetic field varying as $\mathbf{B}_{\mathrm{loc}}(\mathbf{r}) \approx \mathbf{B} + \bar{\bar{G}} \cdot \mathbf{r} + \cdots$. Here $\mathbf{B}$ denotes the uniform applied bias field $\mathbf{B} = B\cos\theta \mathbf{e}_x + B\sin\theta \mathbf{e}_y$, where the angle θ and modulus B are determined by the relative contributions of the $X-$ and $Y-$ bias coils in Fig. 3**a**. The magnetic gradient $\bar{\bar{G}} = (\nabla \otimes \mathbf{B}_{\mathrm{loc}})_{\mathbf{r}=0}$ is in general a rank-2 tensor obeying $\sum_i \bar{\bar{G}}_{ii} = 0$. Atoms in a quantum gas experiment are slow enough to follow adiabatically the direction of the local magnetic field B_{loc} as they move in space. Equivalently, atoms in the Zeeman state $|m_F\rangle$ experience an adiabatic (Born-Oppenheimer) magnetic potential of the form $V_{m_F} = g_F m_F \mu_B |\mathbf{B}_{\mathrm{loc}}(\mathbf{r})|$, where $g_F = -1/2$ is the Landé $g-$factor and μ_B is the Bohr magnetonwhere. The modulus of the local magnetic field is given by $|\mathbf{B}_{\mathrm{loc}}(\mathbf{r})| \approx B + \sum_i 2B_i \bar{\bar{G}}_{ij} r_j + \cdots$.

Residual magnetic forces along y, x are negligible due to the larger confinement in these directions. The relevant contribution of the local magnetic field is then safely approximated by

$$|\mathbf{B}_{\mathrm{loc}}(\mathbf{r})| \approx B + 2\left(\cos(\theta)\bar{\bar{G}}_{xx} + \sin(\theta)\bar{\bar{G}}_{yx}\right)x + \cdots \quad (1)$$

Stray magnetic field gradients contributing to $\bar{\bar{G}}_{ix}$ have at least two origins: (*i.*) the residual ambient gradients (arising from inhomogeneously magnetized elements around the experiment, power supplies, etc ...) and (*ii.*) the imperfections of the bias coils that produce slightly inhomogeneous fields instead of the intended uniform $\mathbf{B}$. We expect that the contribution of *(ii.)* to $\bar{\bar{G}}$ is proportional to the applied bias, since both are created by the same coil system. As a result, the effective magnetic force along x is proportional to $G^{(i)}(\theta) + G^{(ii)}(\theta)B$ where $G^{(i)}, G^{(ii)}$ depend on the orientation of $\mathbf{B}$ but not on its modulus.

In order to compensate the on-axis gradients, we use two separate methods. The first method is to compensate directly the magnetic force by applying an additional weak gradient b' along the x axis to achieve $G^{(i)}(\theta) + G^{(ii)}(\theta)B + b' = 0$. This direct method can become unsuitable when B increases and the maximum achievable gradient b' is too small to compensate for the imperfections of the coils (term $G^{(ii)}(\theta)B$). A second compensation method consists in finding the orientation of $\boldsymbol{B}$ in the $x-y$ plane that cancels the gradients on-axis, *i.e.* finding θ^* such that $G^{(i)}(\theta^*) + G^{(ii)}(\theta^*)B = 0$. None of the two compensation methods is practical in the entire range of bias fields explored in this work. On one hand, the angle tuning method does not work for low bias fields where the contribution from ambient gradients $G^{(i)}$ is too large compared to the imperfections of the bias coils. On the other hand, the direct compensation method is impractical in our experimental setup for bias fields $B \geq 70$ mG, where the sign of the compensation gradient changes (see Supplementary Figure 3**d**). As a result, we combine both methods in the experiments presented in the main article, using the direct compensation method for $B < 70$ mG and the angle-tuning method for $B \geq 70$ mG.

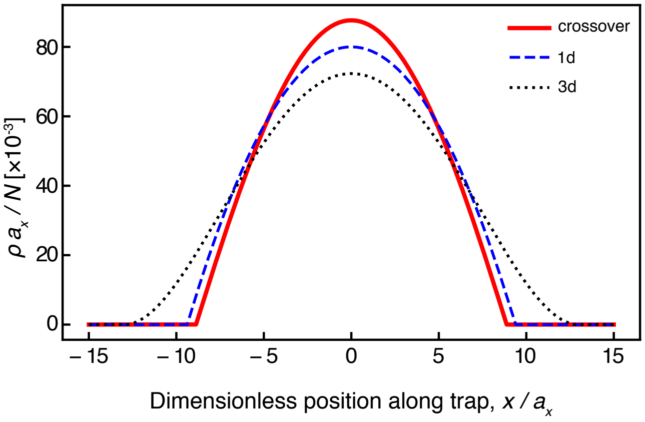

SUPPLEMENTARY FIGURE 4. **Calculated density profiles in the 1d-3d crossover**. Density profile for $N = 10^4$ atoms, $(\omega_x, \omega_y, \omega_z) = 2\pi \times (5.4, 480, 480)$ Hz [$\chi \approx 0.328$ and $\alpha \approx 0.887$].

In order to identify the proper magnetic field configuration, we prepare spin domains with $m_z \approx 0.5$ in a static bias field with amplitude $B \in (10, 600)$ mG. We scan the compensation gradient b'_x with fixed orientation $\theta = 78°$ (Supplementary Figure 3**b**), or the angle θ with $b'_x = 0$ (Supplementary Fig. 3**c**). In both cases we measure the displacement of the Zeeman components as a function of b'_x or θ. The compensation point is then determined by looking for identical c.o.m. for all Zeeman components (correcting for the Stern-Gerlach trajectories which are independently calibrated).

Dimensional crossover from one to three dimensions: Our experiments are performed in a regime of dimensional crossover between one and three dimensions with a chemical potential $\mu \lesssim 0.5\hbar\omega_y$. For a single-component gas, the linear density profile ρ_{1d} is well approximated by [3],

$$\rho_{1\mathrm{d}}(x) = \frac{\alpha\Upsilon(1-\tilde{x}^2)\left(\alpha\Upsilon(1-\tilde{x}^2)+4\right)}{16a}. \quad (2)$$

Here $\Upsilon(x) = x\Theta(x)$, Θ is the Heaviside step function with $\tilde{x} = x/L$. The parameter α is linked to the chemical potential $\mu = \hbar\omega_y(1+\alpha/2)$ (note the origin of energies, different from the choice made in the main text) and to the characteristic length $L = a_x^2\alpha^{1/2}/a_y$, with

$a_{x,y} = \sqrt{\hbar/(m_{\rm Na}\omega_{x,y})}$ the harmonic oscillator lengths along the strong and weak axis of the trap. For a total atom number N, α solves $\alpha^3(\alpha+5)^2 = (15N\bar{a}a_y/a_x^2)^2$. Supplementary Figure 4 compares the density profiles predicted by this approach or in the pure 1d limit ($\alpha \to 0$) for our experimental parameters. The peak density and cloud width in the dimensional crossover are slighly below the pure 1d predictions, but the differences are small.

For a spin-1 system, the stricly 1d approach of [4] can be generalized to include the dimensional crossover corrections. Ref. [4] assumes that the total density is given by the 1d Thomas-Fermi profile of a single-component gas, and proceeds to deduce the expression for q_1 and q_2 given in the main article. This description is valid to lowest order in the small parameter $g_s/\bar{g} \approx 0.036$. Following the same reasoning but using the density profile in supplementary Eq. (2) instead of the strictly 1d Thomas-Fermi profile, one can obtain the values of the critical fields q_1 and q_2 including the dimensional crossover corrections. These values differ slightly from the purely 1d predictions, roughly in the same ratio as the peak densities (less than 10 %, see Supplementary Figure 4). The deviations from the strict 1d limit cannot explain the large difference between the measured q_1 and the expected one.

Magnetic phase diagram: Additional data – Supplementary Figure 5 shows additional data on the magnetic phase diagram.

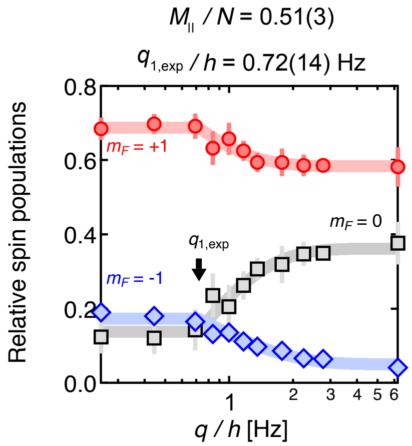

SUPPLEMENTARY FIGURE 5. **Magnetic transition for $M_{||}/N = 0.51(3)$.** Relative populations of the Zeeman states for the magnetic phase diagram shown in Fig. 2 of the main text.

SUPPLEMENTARY REFERENCES

[1] F. Chevy, V. Bretin, P. Rosenbusch, K. W. Madison, and J. Dalibard, "Transverse breathing mode of an elongated bose-einstein condensate," Phys. Rev. Lett. **88**, 250402 (2002).

[2] T. Zibold, V. Corre, C. Frapolli, A. Invernizzi, J. Dalibard, and F. Gerbier, "Spin-nematic order in antiferromagnetic spinor condensates," Phys. Rev. A **93**, 023614 (2016).

[3] F. Gerbier, "Quasi-1d bose-einstein condensates in the dimensional crossover regime," EPL (Europhysics Letters) **66**, 771 (2004).

[4] Tomasz Świsłocki, Emilia Witkowska, Jacek Dziarmaga, and Michał Matuszewski, "Double universality of a quantum phase transition in spinor condensates: Modification of the Kibble-Zurek mechanism by a conservation law," Phys. Rev. Lett. **110**, 045303 (2013).
